# A Case Report of Primary Malignant Melanoma of the Gallbladder with Multiple Metastases

**DOI:** 10.1155/2023/4847053

**Published:** 2023-12-12

**Authors:** Steven H. Adams, Erinn Luo, Daniel Lozeau, Xiaoyun Wen

**Affiliations:** ^1^Stony Brook Medicine Department of Pathology, Stony Brook, New York, USA; ^2^Ward Melville High School, East Setauket, New York, USA; ^3^Stony Brook Medicine Department of Surgery, Stony Brook, New York, USA

## Abstract

Primary malignant melanoma of the gallbladder is an extremely rare tumor with approximately 39 cases described in the literature so far. However, since the first case was reported in 1907, it remains controversial whether gallbladder involvement in malignant melanoma is primary or metastatic. Here, we report a case of primary malignant melanoma of the gallbladder. A 52-year-old male presented to the emergency department with right upper quadrant abdominal pain and was found to have tumefactive sludge filling the majority of the gallbladder with possible gallbladder wall thickening on ultrasonography. A laparoscopic cholecystectomy was performed for presumed acute cholecystitis. Histopathologic examination of the gallbladder revealed malignant melanoma arising from the mucosa of the gallbladder. Further clinical investigation excluded other primary sites, supporting a diagnosis of primary malignant melanoma of the gallbladder.

## 1. Introduction

Malignant melanoma remains a fatal disease with high metastatic potential [1]. One of the most common malignancies to metastasize to the gastrointestinal tract is malignant melanoma, with the most common sites including the small bowel (35-65%), followed by the colon (5-9%), and stomach (5-7%) [2–4]. Malignant melanoma metastatic to the gallbladder is very rare and usually accompanied by a widespread metastasis. More rarely, only approximately 39 cases of primary melanoma of the gallbladder have been described [5–13]. Even considering these reported cases, primary gallbladder melanoma remains a controversial subject, and proving its existence as a primary tumor remains a challenge [14, 15]. Here, we present a case of a 52-year-old male without a prior history of melanoma who presented with right upper quadrant abdominal pain and was subsequently diagnosed with primary melanoma of the gallbladder.

## 2. Clinical Presentation

A 52-year-old male presented to the emergency department with a 1-day history of right upper quadrant colicky abdominal pain that became constant with associated nausea, nonbilious, nonbloody vomiting, and subjective fever. The patient had a past medical history of recurrent diverticulitis and gallbladder sludge in addition to a 30-pack-year smoking history. On physical examination, moderate right upper quadrant tenderness was noted. The patient's lab results revealed an elevated white blood cell count of 16.38 K/*μ*L (normal 4.80-10.80 K/*μ*L) with a left shift and normal liver function tests: serum aspartate aminotransferase 33 IU/L (normal < 40 IU/L), serum alanine aminotransferase 41 IU/L (normal < 41 IU/L), serum alkaline phosphatase 78 IU/L (normal 39-117 IU/L), and serum total bilirubin 0.3 mg/dL (normal < 1.2 mg/dL). A right upper quadrant abdominal ultrasound demonstrated tumefactive sludge filling the majority of the gallbladder with questionable gallbladder wall thickening (measuring up to 4 mm) and a positive sonographic Murphy sign compatible with acute cholecystitis in the appropriate clinical setting. A diagnosis of cholecystitis was strongly suggested, so the patient subsequently underwent an uncomplicated laparoscopic cholecystectomy. The gallbladder was found to be acutely inflamed with wall thickening. The patient did not experience any surgical problems after laparoscopic cholecystectomy surgery and was discharged on the postoperative first day.

## 3. Histopathology

**Figure 1:**
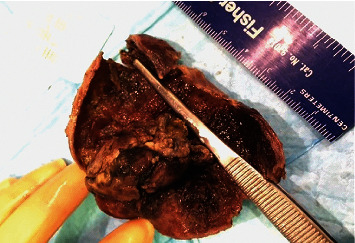
A yellow-brown, friable intraluminal polypoid mass in the gallbladder.

**Figure 2:**
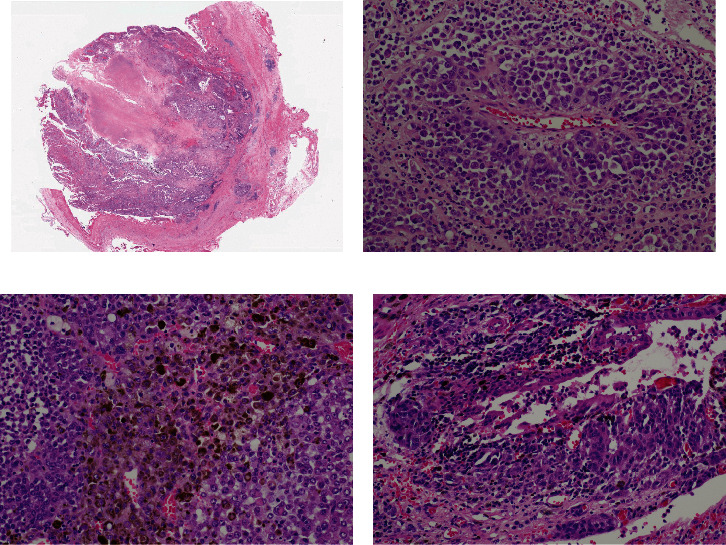
Histological appearance of melanoma of gallbladder. (a) The intraluminal tumor was predominantly arising from the mucosa of the gallbladder (HE × 20). (b) The tumor is composed of polygonal cells arranged in a perivascular pattern with abundant eosinophilic cytoplasm, vesicular nuclei, and prominent eosinophilic nucleoli. (c) Dense melanin pigments were found within the cytoplasm of the neoplastic cells (HE × 200). (d) Junctional activity was present (HE × 200).

**Figure 3:**
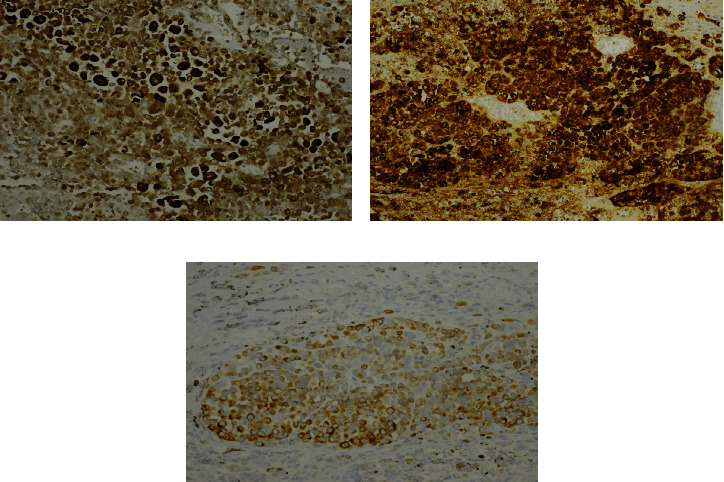
Neoplastic cells were immunostained with anti-S-100 (a), HMB-45 (b), and MART-1 (c) antibodies. ×200.

Macroscopic examination of the gallbladder revealed a 3.9 × 2.5 × 2.0 cm yellow-brown, friable intraluminal polypoid mass in the gallbladder (Figure 1). Histopathologic examination of the gallbladder tumor showed that the intraluminal tumor was predominantly arising from the mucosa of the gallbladder (Figure 2(a)). The tumor was composed of nests of large atypical epithelioid cells arranged in a perivascular pattern with abundant eosinophilic cytoplasm, pleomorphic vesicular nuclei, and prominent nucleoli in a background of acute and chronic inflammation (Figure 2(b)). Occasional intranuclear pseudoinclusions were noted. Additionally, the tumor cell cytoplasm contained abundant granular brown pigment (Figure 2(c)). Junctional activity was identified, featuring aggregates of malignant melanoma cells at the junction of epithelium and lamina propria (Figure 2(d)). The tumor focally invaded the muscularis layer. Immunohistochemical stains showed that the tumor cells were positive for S-100 (Figure 3(a)), HMB-45 (Figure 3(b)), and MART-1 (Figure 3(c)). Molecular analysis revealed the absence of KIT mutations, NRAS mutations, or the BRAF codon 600 mutation in the tumor. The patient was diagnosed with malignant melanoma of the gallbladder. Subsequent investigations were performed to determine whether malignant melanoma of the gallbladder was primary or metastatic, including detailed physical examination, ophthalmologic and dermatologic examinations, colonoscopic examination, and positron emission tomography. The patient was diagnosed with primary malignant melanoma of the gallbladder since no other primary focus of the disease was identified. After surgery, the patient received chemoimmunotherapy with pembrolizumab for 4 cycles without significant side effects.

## 4. Clinical Follow-Up

Approximately nine months after the cholecystectomy, the biopsies of multiple lung nodules revealed the development of pulmonary metastases of malignant melanoma, and subsequent abdominal computed tomography (CT) with contrast revealed a new metastatic lesion on the anterior surface of the liver at the junction of the right and left lobes. Two months later, multiple additional peritoneal metastatic lesions were detected by CT. The patient then transferred care to another institution and was lost to follow-up.

## 5. Discussion

Primary malignant melanoma of the gallbladder is an extremely rare tumor. Since the first case was reported in 1907 [6], it is still controversial whether this disease really exists, and proving its existence as a primary tumor remains a challenge.

Criteria to establish the gallbladder as a primary site for malignant melanoma have been suggested and include (a) the histologic presence of melanoma cells at the junction of epithelium and lamina propria, “junctional activity,” in the setting of a (b) solitary, (c) polypoid or papillary tumor within the gallbladder lumen, with (d) the absence of synchronous involvement at other sites, and (e) the exclusion of other possible primary locations [7, 8, 10]. The latter point requires a thorough medical history and clinical exam including dermatologic, ophthalmologic, and radiologic examinations. The case we reported here met all five criteria and was therefore diagnosed as primary gallbladder melanoma with multiple metastases.

Adrian et al. [8] identified 36 cases of primary gallbladder melanoma reported between 1907 and 2017. Of these, only approximately one-quarter fulfilled all five criteria for establishing the gallbladder as the primary site, while the remaining cases lacked one or more of these criteria.

Since the review by Adrian et al. [8]^,^ there have been three further reports of primary gallbladder melanoma [11–13]. The case of Wang et al. [11] failed to fulfill criterion (d) of absence of synchronous involvement at other sites. In the case of Jeon et al.'s [13] criterion, (a) junctional activity could not be assessed. These limitations demonstrate the difficulty in firmly establishing the gallbladder as a primary site for melanoma. Because of the possibility of a regressed nongallbladder primary tumor site, some have questioned whether primary melanoma of the gallbladder actually exists at all [14, 15]. However, as melanocytes can populate the normal gallbladder, a primary melanoma arising from the gallbladder is at least theoretically possible [16].

Amongst the cases reported thus far, the ratio of male to female is roughly equal, with a median age of approximately 50 years. In the review by Adrian et al. [8], approximately two-thirds of the reported cases presented with symptoms of acute cholecystitis. The vast majority (84.0%) of cases described epithelioid morphology, while a minority of cases (33.3%) showed an additional or exclusive sarcomatoid pattern.

In the modern age of molecular pathology, it may be wise to reconsider and expand the criteria for establishing the gallbladder as the primary site. Recent studies have demonstrated that melanomas of mucosal tissue (gastrointestinal tract, genitourinary tract, and head and neck) are associated with a lower prevalence of BRAF and NRAS mutations compared to cutaneous melanomas. The absence of these mutations in our patient further supports a mucosal (gallbladder) primary over a cutaneous primary. However, some have found an increased prevalence of KIT mutations in mucosal melanomas over cutaneous melanomas [14]. KIT mutations were absent in our patient.

Here is presented a rare case of presumed primary malignant melanoma of the gallbladder. This site of origin is supported grossly by a solitary polypoid lesion, histologically by junctional activity, clinically by thorough patient examination to exclude other primary sites, and molecularly by the absence of BRAF and NRAS mutations.
